# The association between early formula and reduced risk of cow’s milk allergy during the first three year of life: a Japanese cohort study

**DOI:** 10.1186/s13223-022-00712-z

**Published:** 2022-08-07

**Authors:** Kota Ikari, Junichiro Tezuka, Masafumi Sanefuji, Jiro Nakayama, Daisuke Nishima, Yuri Sonoda, Masanobu Ogawa, Masayuki Shimono, Reiko Suga, Satoshi Honjo, Koichi Kusuhara, Shouichi Ohga, The Japan Environment and Children’s Study (JECS) Group, Michihiro Kamijima, Shin Yamazaki, Yukihiro Ohya, Reiko Kishi, Nobuo Yaegashi, Koichi Hashimoto, Chisato Mori, Shuichi Ito, Zentaro Yamagata, Hidekuni Inadera, Takeo Nakayama, Hiroyasu Iso, Masayuki Shima, Youichi Kurozawa, Narufumi Suganuma, Koichi Kusuhara, Takahiko Katoh

**Affiliations:** 1 Division of Allergy and Pulmonology, Fukuoka Children’s Hospital, Fukuoka, Japan; 2 Department of Pediatrics, Faculty of Medicine, Saga University, 5-1-1 Nabashima, Saga, 849-8501 Japan; 3 Department of Pediatrics, Graduate School of Medical Sciences, Kyushu University, Fukuoka, Japan; 4 Department of Bioscience and Biotechnology, Faculty of Agriculture, Graduate School, Kyushu University, Fukuoka, Japan; 5 Research Center for Environment and Developmental Medical Sciences, Kyushu University, Fukuoka, Japan; 6 Department of Obstetrics and Gynecology, Graduate School of Medical Sciences, Kyushu University, Fukuoka, Japan; 7 Department of Pediatrics, University of Occupational and Environmental Health, Kitakyushu, Japan; 8 Regional Center for Japan Environment and Children’s Study, University of Occupational and Environmental Health, Kitakyushu, Japan; 9 Department of Pediatrics, National Hospital Organization Fukuoka National Hospital, Fukuoka, Japan

**Keywords:** Child, Cow’s milk, Food allergy, Observational study, Questionnaire survey

## Abstract

**Background:**

Our recent observational study showed that regular consumption of cow’s milk (CM) formula during early infancy (3–6 months old) was associated with a reduced risk of CM allergy (CMA) at 12 months old. However, the long-term association is unclear. The present study was aimed to examine how long this inverse association persists after 12 months old.

**Methods:**

This study used the dataset of an ongoing nationwide prospective cohort, the Japan Environment and Children’s Study, in which participants were registered between January 2011 and March 2014. We analyzed 65,568 children followed-up until 36 months old. The exposure factors were the consumption statuses of formula milk from 0–3, 3–6, and 6–12 months old. The primary outcome was the prevalence of CMA at 6, 12, 18, 24 and 36 months old. CMA was defined as an allergic reaction and sensitization to CM protein in an individual with no or limited intake of this protein at the evaluation time, combined with physician-diagnosed food allergy. Multivariable regression models were used to estimate the association between the periods of formula consumption and the prevalence of CMA.

**Results:**

The prevalence of CMA increased with a peak of 1.51% at 18 months old and then declined to 0.79% at 36 months old. Formula milk from 3–6 months old was associated with a reduced risk of CMA throughout the first 3 years of life, although the extent of the reduction was mitigated with age (adjusted relative risk: [95% confidence interval]: 0.19 [0.10–0.34] at 12 months old, 0.23 [0.16–0.33] at 18 months old, 0.41 [0.26–0.64] at 24 months old, and 0.47 [0.26–0.80] at 36 months old). The association between early formula and CMA were observed in both children with and without eczema, but more prominent and long-lasting in the former than the latter.

**Conclusions:**

Regular exposure to CM protein during infancy was associated with a reduced prevalence of CMA during early childhood. At present, however, this observational study does not necessarily encourage formula feeding, and randomized controlled trials are warranted to confirm the findings and their significance.

**Supplementary Information:**

The online version contains supplementary material available at 10.1186/s13223-022-00712-z.

## Background

Cow’s milk allergy (CMA) is one of the most common food allergies [1] but often resolves in early childhood. Although breastfeeding has many benefits and is thus recommended for all infants, cow’s milk (CM) formula can be used as a substitute for breastmilk. Observational studies have demonstrated that a reduced risk of CMA is associated with regular consumption of formula started by three months old [2–5] whereas others have shown no definite association [6]. A recent randomized controlled trial revealed that regular consumption of CM formula in early infancy prevents the CMA development at six months old [7], strongly supporting the protective effect of early CM protein on CMA, as is observed in cases with peanut and hen’s egg allergy [8, 9]. However, little has been reported concerning the long-term effects of early CM protein ingestion.

Our recent study reported that the risk of CMA at 12 months old was reduced when regular consumption of CM formula was introduced within the first 3 months of age [10]. In the study, we further demonstrated that the reduced risk was actually associated with formula consumption in early infancy (3–6 months old) rather than very early infancy (0–3 months old), suggesting the effect of very early CM exposure on CMA may disappear if the exposure is brief. This result was derived from the data obtained in children under 12 months old in the Japan Environment and Children’s Study (JECS), which released a new dataset containing data for the first 3 years of life. The goal of the present study was to examine whether or not the reduced risk of CMA associated with early formula ingestion persisted after 12 months old. The results may help allergists and pediatricians predict the occurrence of CMA depending on the period of formula feeding, to facilitate personalized management of this allergy.

## Methods

### Study design and participants

The JECS is a nationwide, multicenter, prospective birth cohort study and the details of the study design were reported previously [11, 12]. In brief, pregnant participants and their partners were registered between January 2011 and March 2014 at 15 research sites covering a wide geographical area of Japan. Information was mainly obtained from medical record transcripts and self-reported questionnaires. The JECS was conducted in accordance with the Declaration of Helsinki. The protocol was reviewed and approved by the Ministry of Environment’s Institutional Review Board for Epidemiological Studies and by the Ethics Committees of all the participating institutions (#100910001). Written informed consent was obtained from all participants.

### Participants

**Fig. 1:**
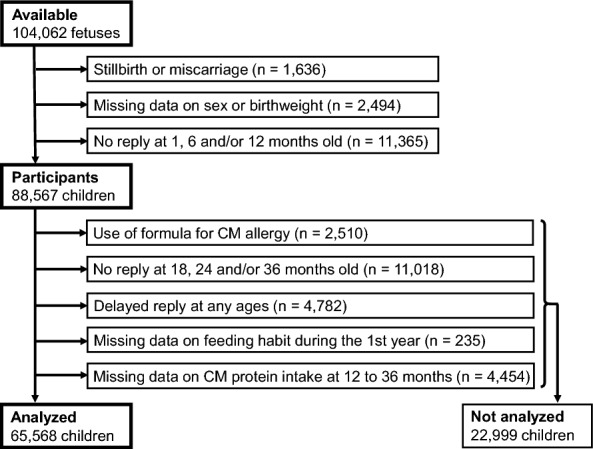
Flow chart for participant selection. CM, cow’s milk

In this study, we used the fixed dataset “jecs-ta-20190930”, which was released in October 2019. The dataset contains the data for 104,062 fetuses from 103,060 pregnancies until the child reached 36 months old. A total of 88,567 live-born children for whom information on sex and birthweight had been recorded and for whom data were available at 1, 6 and 12 months old were selected as participants (Fig. 1). Of these children, we excluded those who used formula specialized for CMA (e.g. extensively-hydrolyzed formula), whose reply was absent at 18, 24 and/or 36 months old and whose reply was delayed at 6, 12, 18, 24 or 36 months old. A questionnaire was regarded as ‘delayed’ when a response was received over 2 months after delivery of 6- and 12-month questionnaires and over 3 months after delivery of 18-, 24- and 36-month questionnaires. We also excluded children who had missing information on feeding habit during the 1 year of life or intake statuses of CM protein at any ages of 12 to 36 months old. After excluding these 22,999 children, data for 65,568 children remained for the analysis.

### Exposure

The main exposure factor was whether a child regularly consumed CM formula during the 1 year of life, divided into 3 periods: very-early (0–3 months old), early (3–6 months old) and late infancy (6–12 months old), as reported previously [10]. In each period, an infant was defined as consuming substantially formula milk if he or she received formula milk over half of the period (for 2 or 3 months during the 3-month period of very-early and early infancy; for 4, 5 or 6 months during the 6-month period of late infancy). The validity and reliability of this definition was confirmed in the previous study [10]. Before 12 months old, unfortunately, the JECS only collected data concerning the commencement timing of dairy food but not the detailed consumption status. Since our previous study demonstrated that the main results were similar between the analyses between before and after excluding the participants who had commenced dairy foods [10], this information was not analyzed in the current study.

### Outcome

The primary outcome was the prevalence of CMA at 6, 12, 18, 24 and 36 months old. Inclusion criteria for CMA at 6 and 12 months old were (1) parent-reported allergic reactions of the child to CM protein, such as formula milk and dairy food; (2) no consumption of CM protein at the evaluation time; and (3) a physician’s diagnosis of food allergy, as reported previously [10]. The same criteria were used for CMA at 18, 24 and 36 months old, but limited consumption of CM protein was also acceptable under the (2) criterion. At these older ages, an additional criterion was (4) positivity of serum-specific IgE or positive skin-prick test to CM protein in clinics or hospitals, although the values of the former and the wheal sizes of the latter were unknown. Information on these criteria was obtained from the questionnaire at each age. A child was regarded as having CMA when they met all criteria at that evaluation time.

### Statistical analyses

To examine the association between the periods of formula and CMA, we used logistic regression models. The regression models included the three periods of formula, as well as covariates, determining the impact of each period independently from the influence of other periods [10]. The covariates included i) sex; ii) mode of delivery (vaginal vs. Caesarean); iii) any maternal allergic disease; iv) maternal smoking during pregnancy, confirmed in the first trimester; v) maternal education level (junior high school, high school, or university or graduate school); vi) annual family income (low: < 4,000,000; middle: 4,000,000–5,999,999; high: ≥ 6,000,000 Japanese yen); vii) early eczema, occurring within the first 3 months of life and defined as an intermittent itchy rash over a period of ≥ 2 months; and viii) living with older siblings. As covariates, we also included ix) the intake statuses of CM proteins other than formula milk (e.g. pasteurized milk, cheese, etc.) before that evaluation time (e.g. in estimating the risk of CMA at 24 months old, the intake statuses of CM proteins at 12 and 18 months old were included in the model). The information on the statuses was collected using the 12-, 18-, 24- and 36-month questionnaires, which asked whether a child ordinarily consumed the proteins (versus no or limited intake) at that time. Statistical analyses were performed using the R software program, version 4.0.3. Firth’s bias reduction method was employed when separation occurred in a logistic regression analysis, using ‘logistf’ version 1.23 in the R package. We reported the adjusted relative risks (aRRs) with 95% confidence intervals (CIs) that were converted from odds ratios in an established method. [13, 14]

## Results

### Study population

The baseline characteristics of the 65,568 children included in our analysis are shown in Table 1. Allergic disease was recorded in 55.3% of mothers, and early eczema was observed in 24.2% of children. At 6 and 12 months old, CMA was observed in 150 (0.23%) and 682 (1.04%) children, respectively. These prevalences were similar to those in our previous study that had included 80,408 children in the analysis [10]. The 22,999 children excluded from the analysis showed comparable prevalences of CMA to 65,568 children for the analysis. However, they tended to show maternal characteristics of a younger age, more frequent smoking habit and lower education and income as well as child’s characteristics of higher rates of living with older siblings and formula use.

**Table 1:** Baseline characteristics of the participants (n = 88,567)

	Analyzed (n = 65,568)	[Missing]	Not analyzed (n = 22,999)	[Missing]	Effect size^a^
Males (%)	33,416 (51.0)	0	11,990 (52.1)	0	0.01
Gestational age, weeks (SD)	39.2 (1.6)	0	39.2 (1.7)	0	0.02
Birthweight, g (SD)	3,011 (418)	0	3,021 (439)	0	0.01
Maternal age, years (SD)	31.6 (4.8)	0	30.7 (5.2)	4	0.08
Caesarean section (%)	12,771 (19.5)	135	4,697 (20.5)	43	0.01
Maternal allergic diseases (%)	36,113 (55.3)	245	12,387 (54.3)	183	0.01
Maternal smoking during pregnancy (%)	9,357 (14.4)	618	4,977 (22.0)	393	0.09
Maternal education		480		430	0.09
Junior high school (%)	2,135 (3.3)		1,422 (6.3)		
High school (%)	47,166 (72.5)		17,161 (76.0)		
University/graduate school (%)	15,787 (24.3)		3,986 (17.7)		
Family income		4,251		2,131	0.07
Low (%)	22,822 (37.2)		9,385 (45.0)		
Middle (%)	20,996 (34.2)		6,521 (31.2)		
High (%)	17,499 (28.5)		4,962 (23.8)		
Early eczema (%)	15,596 (24.2)	1,169	5,282 (23.5)	492	0.01
Older siblings (%)	34,737 (53.2)	245	13,175 (57.7)	183	0.04
Formula consumption (%)					
Very early (0–3 months old)	30,540 (46.6)	0	11,806 (52.0)	315	0.05
Early (3–6 months old)	26,567 (40.5)	0	11,525 (50.1)	0	0.08
Late (6–12 months old)	28,331 (43.2)	0	12,259 (53.3)	0	0.09
Prevalence of food allergy (%)					
At 6 months old					
CMA	150 (0.2)	0	80 (0.3)	0	0.01
At 12 months old					
CMA	682 (1.0)	0	229 (1.0)	4	0.00

*CMA* cow's milk allergy, *SD* standard deviation

^a^Effect size indicates the strength of difference between groups and are calculated as *phi*/Cramer's *V* and *r* using the chi-square and Student's *t*-tests for categorical and numerical variables, respectively

### Formula administration periods and food allergies

**Fig. 2:**
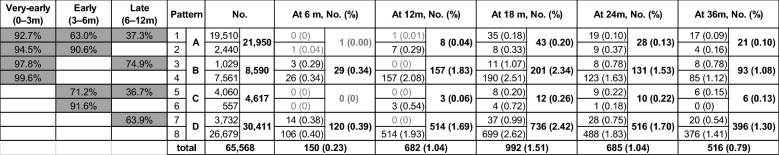
Patterns of formula feeding and the prevalence of cow’s milk allergy (CMA). Gray and white boxes denote consumption and non-consumption, respectively, of formula milk. The number in the gray boxes represents the percentage of subjects with concurrent use of breastmilk among formula-fed children. For each pattern, the prevalences of CMA are provided. The prevalence in the grayed areas is zero or nearly zero, as expected from the definition of CMA, which required no consumption of CM products including formula at the evaluation times

To overview the relationship between formula consumption and CMA, we classified the participants into 8 patterns (patterns 1–8) according to the formula statuses of the three periods (very-early [0–3 months], early [3–6 months], and late periods [6–12 months]), and calculated the prevalence of CMA for each pattern (Fig. 2), as analyzed similarly in our previous study [10]. Since the study had revealed the major contribution of formula in the early period to the risk of CMA at 12 months old, the participants were further classified into 4 patterns (patterns A-D) according to the ingestion in the very-early and early periods. The overall prevalence of CMA increased with age, peaking at 18 months old (992 children, 1.51%) and then declining at 36 months old (516, 0.79%). As we reported previously [10], the prevalence of CMA at 12 months old was lower when children consumed formula in the early period (0.04% and 0.06% for patterns A and C) than when they did not (1.83% and 1.69% for patterns B and D). The reduced prevalence of CMA in the early formula group continued until 3 years old.

Multivariable regression models were used to estimate the association between the period of formula consumption (versus no consumption) and the prevalence of CMA (Table 2a). Early formula ingestion was consistently associated with a reduced risk of CMA throughout the first 3 years of age, although the extents of the reduced aRRs were mitigated as the children aged (0.19, 0.23, 0.41 and 0.47 from 12, 18, 24 and 36 months old, respectively), with no CIs crossing 1. In addition, the late formula was associated with a reduced risk of CMA at 18 and 24 months old (aRR: 0.42 and 0.61, respectively), although the extent of the association was lower than those for early formula ingestion. In contrast, very-early formula ingestion had no association with the risk of CMA at any age.

**Table 2:** The association between periods of formula consumption and food allergies

a. All children for the analysis (n = 59,560)					
	CMA				
	At 6 m	At 12 m	At 18 m	At 24 m	At 36 m
Formula consumption	aRR (95% CI)	aRR (95% CI)	aRR (95% CI)	aRR (95% CI)	aRR (95% CI)
Very early (0–3 months old)	1.00 (0.65–1.51)	1.14 (0.94–1.36)	1.10 (0.92–1.30)	1.08 (0.86–1.35)	0.97 (0.73–1.27)
Early (3–6 months old)	0.01 (0.00–0.05)^a^	**0.19 (0.10–0.34)**	**0.23 (0.16–0.33)**	**0.41 (0.26–0.64)**	**0.47 (0.26–0.80)**
Late (6–12 months old)	**–**	0.01 (0.00–0.03)^a^	**0.42 (0.32–0.55)**	**0.61 (0.42–0.87)**	0.96 (0.62–1.45)

b. Exclusion of children who already had allergy by the previous evaluation time					
	CMA				
	At 6 m (n = 59,560)	At 12 m (n = 59,422)	At 18 m (n = 58,855)	At 24 m (n = 58,326)	At 36 m (n = 58,195)
Formula consumption	aRR (95% CI)	aRR (95% CI)	aRR (95% CI)	aRR (95% CI)	aRR (95% CI)
Very early (0–3 months old)	1.00 (0.65–1.51)	1.15 (0.94–1.39)	0.82 (0.65–1.03)	1.01 (0.62–1.58)	0.59 (0.27–1.20)
Early (3–6 months old)	0.01 (0.00–0.05)^a^	**0.22 (0.11–0.38)**	**0.32 (0.21–0.47)**	0.53 (0.25–1.10)	0.76 (0.29–1.98)
Late (6–12 months old)	–	0.01 (0.00–0.03)^a^	**0.61 (0.44–0.82)**	0.78 (0.41–1.41)	1.36 (0.62–2.82)

The statuses of formula consumption (versus no consumption) and the following covariates were all included together in the model: sex, delivery mode, maternal allergic diseases, smoking and education, family income, early eczema, and older siblings as well as intake statuses of cow's milk protein before the evaluation time. Bold text represents statistical significance (*p* < 0.05)

*aRR* adjusted relative risk, *CI* confidence interval, *CMA* cow's milk allergy

^a^The result was very low as expected from the definition of CMA, which required no consumption of CM products including formula at the evaluation time

However, the observed associations between formula and the reduced risk of CMA may be accounted for by reverse causation, as the exposure factor (i.e. formula milk) and outcome (i.e. CMA) were interdependent. After a child had experienced an allergy to formula at a certain point (e.g. at 4 months old), the mother would likely avoid giving formula and dairy foods to the child for a long time (e.g. even at 18 months old). This avoidance behavior had the potential to produce superficially an inverse association between formula ingestion (e.g. 3–6 months old) and CMA at a later age (e.g. 18 months old). To address this issue, we performed a sensitivity analysis. When examining the association between formula ingestion and CMA at an evaluation timepoint (e.g. 18 months old), we excluded children who had ever experienced CMA by the previous evaluation timepoint (e.g. 12 months old). In this analysis, if some of the remaining children, who never had experienced CMA, newly developed CMA between the previous evaluation time (e.g. 12 months old) and the evaluation time (e.g. 18 months old), the CMA was unlikely attributable to such reverse causation. This stringent analysis also produced a concordant result (Table 2b), although the significance of the association between formula and CMA at 24 and 36 months old disappeared.

### Analyses stratified by eczema

In addition, the observed associations between formula and reduced risks of the food allergies might have been produced artificially by the presence of early eczema. When a child has severe eczema in early infancy, parents may avoid formula feeding, trusting in the benefits of breastmilk. Eczematous children have been reported to be at a high risk of food allergy [15, 16]. Indeed, in our participants, the prevalences of CMA were consistently higher throughout the periods in children with early eczema than in those without (Additional file 1). Thus, formula avoidance by parents of eczematous children who were at a high risk of food allergy might have yielded an artificial inverse association between formula and food allergies.

To address this issue, we stratified the children into groups with and without early eczema and then examined the associations between formula consumption (versus no consumption) and CMA separately for the two groups. As expected, the eczematous children were more likely to have mothers with allergic diseases and less likely to consume formula milk than the non-eczematous children. In regression analyses, associations between early formula ingestion and reduced risks of CMA were observed in both the eczematous and non-eczematous groups, although the associations of the former were more prominent and long-lasting than in the latter (Table 3).

**Table 3:** The eczema-stratified association between periods of formula consumption and CMA

	Eczema (n = 14,451)				
	At 6 m	At 12 m	At 18 m	At 24 m	At 36 m
Formula consumption	aRR (95% CI)	aRR (95% CI)^b^	aRR (95% CI)	aRR (95% CI)	aRR (95% CI)
Very early (0–3 months old)	1.51 (0.92–2.38)	1.15 (0.90–1.44)	1.11 (0.89–1.38)	1.01 (0.75–1.36)	0.95 (0.66–1.35)
Early (3–6 months old)	0.02 (0.00–0.07)^a^	**0.14 (0.05–0.32)**	**0.23 (0.14–0.37)**	**0.36 (0.18–0.68)**	0.43 (0.19–0.94)
Late (6–12 months old)	–	0.01 (0.00–0.04)^a^	**0.56 (0.39–0.78)**	0.74 (0.46–1.16)	0.95 (0.54–1.60)

	No eczema (n = 45,109)				
	At 6 m	At 12 m	At 18 m	At 24 m	At 36 m
Formula consumption	aRR (95% CI)^b^	aRR (95% CI)	aRR (95% CI)	aRR (95% CI)	aRR (95% CI)
Very early (0–3 months old)	**0.37 (0.12–0.88)**	1.13 (0.83–1.51)	1.09 (0.83–1.40)	1.16 (0.82–1.63)	0.99 (0.65–1.50)
Early (3–6 months old)	0.03 (0.00–0.21)^a^	**0.28 (0.12–0.58)**	**0.26 (0.15–0.44)**	0.49 (0.25–0.93)	0.48 (0.21–1.05)
Late (6–12 months old)	–	0.01 (0.00–0.06)^a^	**0.28 (0.17–0.44)**	**0.45 (0.24–0.80)**	0.95 (0.47–1.82)

The statuses of formula consumption (versus no consumption) and the following covariates were all included together in the model: sex, delivery mode, maternal allergic diseases, smoking and education, family income, early eczema, and older siblings as well as intake statuses of cow's milk protein before the evaluation time. Bold text represents statistical significance after adjustment for two comparisons of the stratified group (*p* < 0.025)

*aRR* adjusted relative risk, *CI* confidence interval, *CMA*, cow's milk allergy

^a^The result was very low as expected from the definition of CMA. ^b^Using Firth's bias reduction method to resolve the issue of separation in regression analysis

## Discussion

### Principal study findings

We previously reported that the risk of CMA at 12 months old was reduced when a child regularly consumed CM formula during early infancy (3–6 months old) rather than in the very-early period (0–3 months old) [10]. In the present study, we demonstrated that this finding continued until 36 months old. The risk of CMA was consistently reduced in children fed formula early, although the extent of the reduced risk was mitigated with age. This association between early formula and CMA were observed in both children with and without eczema, but the effect sizes of the association were more prominent and long-lasting in the former than the latter. These observations suggest that early formula would make an important contribution to CMA development persistently during early childhood.

### Study strengths and weaknesses

As we stated previously [10], the strengths of this study include the prospective design, large sample size and detailed information on formula, which determined the patterns of formula consumption during the 1 year of life. Furthermore, this study contained comprehensive information on the outcome, including allergic reactions, sensitization and the intake status concerning CM proteins as well as the physician’s diagnosis, enabling the identification of children with CMA using self-administered questionnaires.

However, the use of questionnaire-based surveys is also a weakness of this study, as described previously [10]. In particular, information on the gold standard for the diagnosis of food allergy, or oral food challenge tests, was lacking. Furthermore, the exact day-by-day feeding status could not be determined from the questionnaires. When mothers used formula only occasionally during a month, the month would be classified as ‘no use’ of formula. If an exclusively breastfed child received formula milk on a certain day during the month and then developed CMA, the infant’s mother would avoid formula feeding for some time. In this situation, the mother would answer ‘the child did not consume formula’ for that month, and the child might be judged as having CMA at subsequent evaluation points if the avoidance of CM protein was continued. As such, a lack of formula consumption might be due to the occurrence of CMA. As shown in Table 2b, however, the association between early formula ingestion and CMA at 12 and 18 months old remained significant even after the exclusion of children who had already had CMA by 6 and 12 months old, respectively. This suggests an influential effect of formula milk on CMA, although the association may still be explained by reverse causation. Even in questionnaire-based surveys, distinction between regular and intermittent ingestion of formula would be needed to more clearly elucidate the impact of formula on CMA. Finally, many more children (n = 22,999) were excluded from the analysis in this study than in our previous study (n = 8,130) [10]. Although the children excluded showed equivalent prevalences of CMA at 12 months old compared to children included in the analysis, the differences in several baseline characteristics between the excluded and included children suggest potential selection bias.

### Interpretation

The present study demonstrated that the early ingestion of CM formula was associated with a persistently reduced prevalence of CMA during the first 3 years of life. This association may be accounted for by the dual-allergen exposure hypothesis, which states that early cutaneous exposure to food protein though a disrupted skin barrier leads to allergic sensitization, whereas early oral exposure to that protein induces tolerance [15, 17]. Induction of oral tolerance in infancy explains well our finding of the close association between formula ingestion in the early period and a reduced risk of later CMA. Of note, this period of 3–6 months old is considered to be a critical time window for oral induction. [18] Cutaneous sensitization also seems to conform with the results of the eczema-stratified analysis. The elevated risk of CMA in children with early eczema might reflect the cutaneous sensitization to CM protein. As shown in Table 3, the association between the early ingestion of formula and the reduced risk of CMA was more prominent and long-lasting in the children with eczema than in those without. Oral tolerance might be particularly effective when cutaneous sensitization is involved in the pathogenesis of food allergy.

The inverse association between the early formula and CMA in our study agrees with the findings of a recent randomized controlled trial on CMA. In that trial, participants were assigned to either ingest at least 10 mL/day of CM formula or avoid it during 1 to 2 months old, although breastfeeding was continued at a similarly rate between the two groups [7]. The ingestion group was much less likely to develop CMA at 6 months old than the avoidance group. This result seemingly demonstrated the importance of formula consumption before three months old. However, during 3–6 months old, the ingestion group actually consumed formula milk more abundantly, frequently and continuously than the avoidance group. Therefore, the preventive effects against CMA in that study might have been due more to formula ingestion in the later ages than in the earlier ages.

## Conclusions

The present study demonstrated that children who regularly consumed formula milk from 3–6 months old showed a marked reduction in the prevalence of CMA consistently during the first 3 years of life. If formula is started during infancy, its continuation or complementary feeding of dairy food such as yogurt may contribute to the reduction of CMA during early childhood. However, the results of this observational study never discourage breastfeeding, which has significant benefits for mothers and children. Future randomized control trials are needed to properly interpret the results of this study.

## Supplementary Information

**Additional file 1: Table S1.** Baseline characteristics of children with early eczema vs. those without

## Data Availability

Data are unsuitable for public deposition due to ethical restrictions and legal framework of Japan. It is prohibited by the Act on the Protection of Personal Information (Act No. 57 of 30 May 2003, amendment on 9 September 2015) to publicly deposit the data containing personal information. Ethical Guidelines for Medical and Health Research Involving Human Subjects enforced by the Japan Ministry of Education, Culture, Sports, Science and Technology and the Ministry of Health, Labour and Welfare also restricts the open sharing of the epidemiologic data. All inquiries about access to data should be sent to: jecs-en@nies.go.jp. The person responsible for handling enquiries sent to this e-mail address is Dr Shoji F. Nakayama, JECS Programme Office, National Institute for Environmental Studies.

## Abbreviations

aRR: Adjusted relative risk
CI: Confidence interval
CM: Cow’s milk
CMA: Cow’s milk allergy
JECS: Japan Environment and Children’s Study
